# Supplementary material for: Spin-neutral currents for spintronics
Source: Nat Commun. 2021 Dec 3;12:7061. doi: 10.1038/s41467-021-26915-3 (PMC8642435; doi:10.1038/s41467-021-26915-3)
Supplement: Supplementary file 1 — Supplementary Information [file 41467_2021_26915_MOESM1_ESM.pdf]

SUPPLEMENTAL MATERIAL

Spin-neutral currents for spintronics

Ding-Fu Shao,[1,*] Shu-Hui Zhang,[2] Ming Li,[1] and Evgeny Y. Tsymbal[1,†]

[1] *Department of Physics and Astronomy & Nebraska Center for Materials and Nanoscience, University of Nebraska, Lincoln, Nebraska 68588-0299, USA*

[2] *College of Mathematics and Physics, Beijing University of Chemical Technology, Beijing 100029, People's Republic of China*

A. Conduction channels of RuO_2 calculated by ATK

Figure S1 shows the conduction channels of bulk RuO_2 in the 2D Brillouin zone calculated by ATK. The results are in excellent agreement with those obtained using the Wannier tight-binding model derived from the VASP band structure (Fig. 2(c) in the main text).

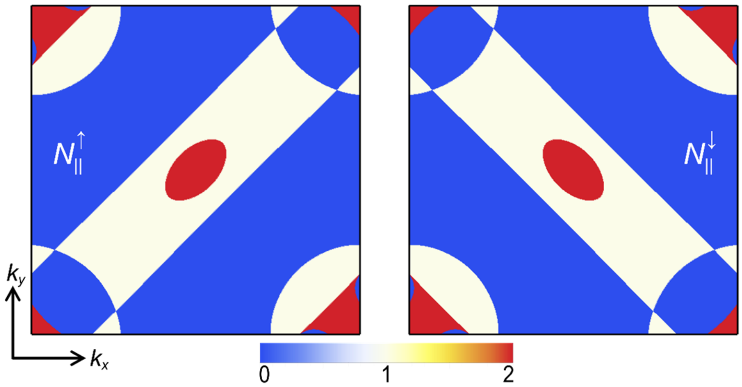

Fig. S1. The conduction channels of bulk RuO_2 in the 2D Brillouin zone calculated using ATK.

B. $RuO_2/TiO_2/RuO_2$ AFMTJ with different TiO_2 thickness

Figures S2(a) and S3(a) show the atomic structures of $RuO_2/TiO_2/RuO_2$ AFMTJs with 7 and 9 TiO_2 monolayers in the barrier, respectively. In these cases, the scattering region has symmetric interfaces with reversed magnetic moment alignment as compared to the AFMTJ with the even number of TiO_2 monolayers shown in Fig. 3(a). As seen from the calculated $\vec{k}_{\parallel}$-resolved transmission in Figures S2 (b) and S3 (b), this enhances the transmission for one spin channel but reduces for the other. However, the total transmission (Figs. S2 (c) and S3 (c)), and TMR (Figs. S2 (d) and S2 (d)) as functions of energy do not change much compared to those shown in Fig. 4, indicating that TMR in $RuO_2/TiO_2/RuO_2$ AFMTJ is robust to interfacial configurations, due to electron transport being controlled by the bulk states of RuO_2.

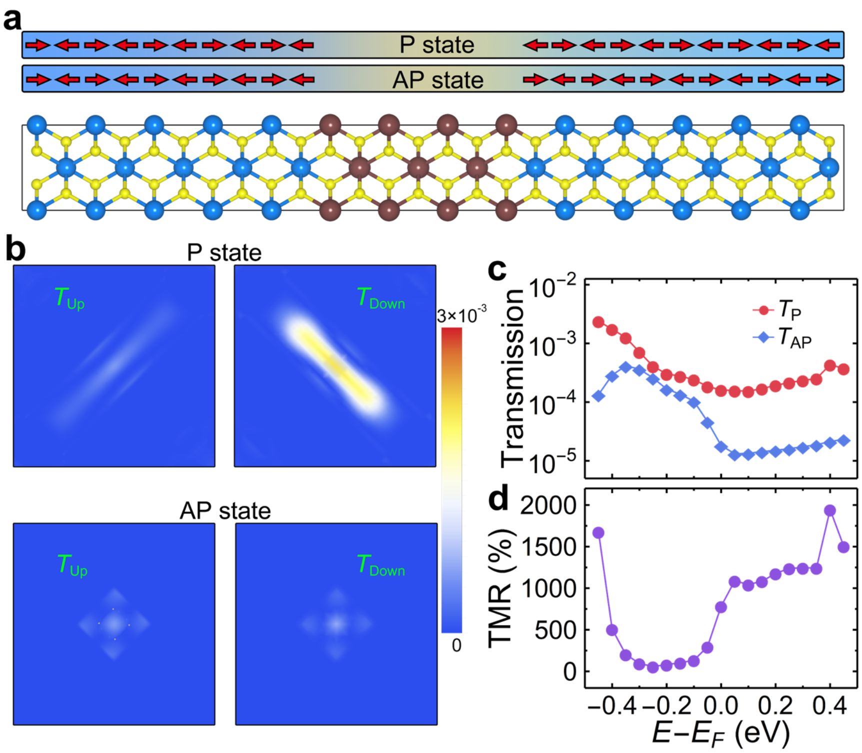

FIG. S2. The atomic structure (a), $\vec{k}_{\parallel}$-resolved transmission in the 2D Brillouin zone (b), transmission as a function of energy (c), and TMR as a function of energy (d) for $RuO_2/TiO_2/RuO_2$ AFMTJ with 7 TiO_2 monolayers.

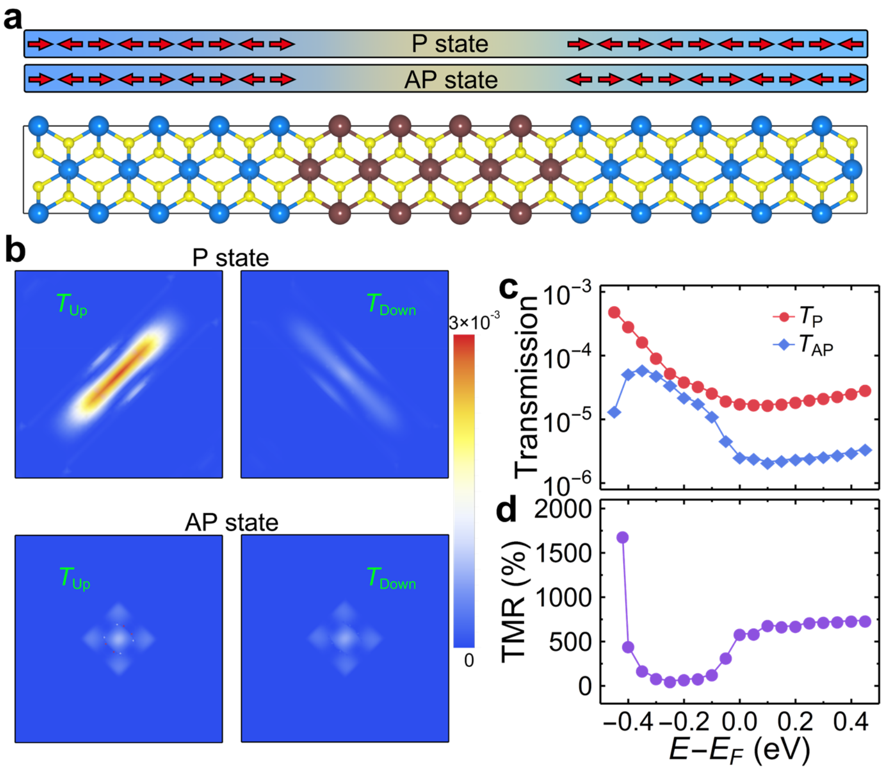

FIG. S3. The atomic structure (a), $\vec{k}_{\parallel}$-resolved transmission in the 2D Brillouin zone (b), transmission as a function of energy (c), and TMR as a function of energy (d) for $RuO_2/TiO_2/RuO_2$ AFMTJ with 9 TiO_2 monolayers.

C. Effects of disorder

In the main text, we calculated the conductance of a $RuO_2/TiO_2/RuO_2$ AFMTJ without considering effects of disorder, which is unavoidable in realistic devices. Although it is difficult to simulate such effects within a first-principles approach, we can model them qualitatively by introducing an effective broadening of the energy levels within the Green's function formalism implemented in the ATK. This is performed using a broadening parameter η in the retarded Green's function G as follows:

$$G(E) = \frac{1}{H - (E + i\eta)S}, \tag{S1}$$

where E is the energy, and S and H are the overlap and Hamiltonian matrices of the entire system, respectively. The state-independent broadening η is related to the inverse lifetime of the carriers produced by disorder.

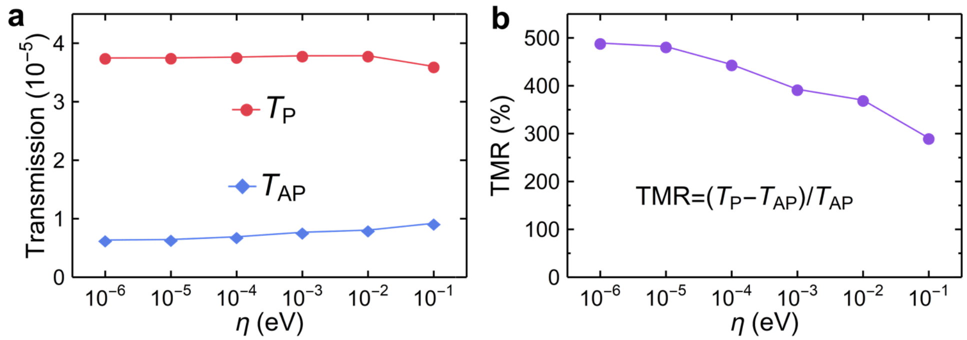

Fig. S4. (a,b) The calculated transmission (a) and TMR (b) as functions of broadening parameter η.

Figure S4 shows the calculated transmission and TMR as functions of η for the $RuO_2/TiO_2/RuO_2$ AFMTJ in Fig. 3. Here, we used a 201×201 $\vec{k}$ -point mesh instead of the adaptive $\vec{k}$ -point mesh to ensure that η is the only variable parameter in the calculation. This led to a minor change in the TMR value for default $\eta = 10^{-6}$ eV (~550% in Fig. 3 and ~490% in Fig. S4) not affecting our main conclusion. We find that the P state transmission (T_P) is nearly a constant for $\eta \leq 0.01$ eV and has a small decrease for a large $\eta = 0.1$ eV. On the other hand, the AP state transmission (T_{AP}) increases monotonically from $\approx 6.4 \times 10^{-6}$ to $\approx 9.2 \times 10^{-6}$ with η changing from 10^{-6} eV to 0.1 eV. This is due to the broadening of the energy levels smearing out the conduction channels and hence reducing their mismatch in the AP state. As a result, the TMR decreases with increasing η. However, we find that the TMR magnitude remains sizable even for η as large as 0.1 eV.

The magnitude of η relevant to the experimental conditions can be estimated by looking at the diffusive resistivity of metals as a function of η. For a metal with moderate disorder, such as RuO_2, we find that the broadening required to reproduce the experimentally measured resistivity is ~ 25−50 meV [1]. For values of η in this energy range, the predicted TMR of the $RuO_2/TiO_2/RuO_2$ AFMTJ is ~350%. These results imply that the predicted TMR is robust with respect to disorder and is expected to be large as long as the crystallinity of the tunnel junction is maintained and a direct tunneling mechanism controls the AFMTJ conductance.

D. Symmetry analysis of the spin polarized conduction channels

The ballistic conductance per unit area of a homogeneous metal along the *z* direction is given by

$$g = \frac{e^2}{h}\int \frac{d^2k_\parallel}{4\pi^2} N_\parallel(\vec{k}_\parallel), \tag{S2}$$

where $N_\parallel(\vec{k}_\parallel)$ is the number of conduction channels at the transverse wave vector $\vec{k}_\parallel = (k_x, k_y)$:

$$N_\parallel(\vec{k}_\parallel) = \frac{1}{2}\sum_n \int \hbar|v_{nz}| \frac{\partial f}{\partial E_n(\vec{k})} dk_z\,. \tag{S3}$$

Here $E_n(\vec{k})$ is energy for the *n*-th band, $v_{nz} = \frac{\partial E_n(\vec{k})}{\hbar \partial k_z}$ is the band velocity along the transport *z* direction, f is the Fermi distribution function. Eqs. (S2) and (S3) are valid in the presence of spin-orbit coupling, in which case the band index *n* implicitly includes the spin index. For a collinear antiferromagnet, in absence of spin-orbit coupling, the spin projection to the Néel vector is a "good" quantum number and Eqs. (S2) and (S3) are reduced to Eqs. (1) and (2) in the main text.

At zero temperature, $\frac{\partial f}{\partial E_n(\vec{k})} = \delta[E_n(\vec{k}) - E_F]$, and the ballistic conductance can be expressed as a purely Fermi surface (FS) property [2]:

$$g = \frac{e^2}{h}\frac{1}{4\pi^2}\frac{1}{2}\sum_n \int_{FS} \frac{dS}{|\vec{v}_n|}|v_{nz}| = \frac{e^2}{h}\frac{1}{4\pi^2}\frac{1}{2}\sum_n S_n(\hat{z}), \tag{S4}$$

where $\vec{v}_n = \frac{1}{\hbar}\nabla_{\vec{k}} E_n(\vec{k})$ is the band velocity and $S_n(\hat{z})$ is the projection of the Fermi surface of band *n* to the transport direction given by the unit vector $\hat{z}$. The number of conduction channels $N_\parallel(\vec{k}_\parallel)$ is determined by the number of roots in the equation $E_n(\vec{k}_\parallel, k_z) = E_F$ summed up over all propagating modes *n*.

Since the number of conduction channels is purely the Fermi surface property, we can analyze their spin polarization by calculating the expectation values of the spin components $\vec{s} = (s_x, s_y, s_z)$ at the Fermi

surface. This allows us to determine the spin polarization of the conduction channels based on the symmetry arguments as explained below.

The time reversal symmetry $\hat{T}$ transforms the energy bands $E_n(\vec{k})$ and the spin $\vec{s}$ as follows:

$$\hat{T}E_n(\vec{k}_\parallel, k_z) = E_n(-\vec{k}_\parallel, -k_z),$$
$$\hat{T}\vec{s} = -\vec{s}\,. \tag{S5}$$

As a result, the net spin polarization of the whole Fermi surface and the net conductance must be zero. Therefore, the spin-polarized current cannot be supported by nonmagnetic materials where $\hat{T}$ is preserved. This is also the case for compensated antiferromagnets where $\hat{P}\hat{T}$ or $\hat{T}\hat{t}$ symmetries are maintained.

In the case of $\hat{P}\hat{T}$ symmetry, not only the net spin polarization, but also of the spin polarization of each conduction channel is zero. This is due to the spin degeneracy of the Fermi surfaces, as seen from

$$\hat{P}\hat{T}E_n(\vec{k}_\parallel, k_z) = E_n(\vec{k}_\parallel, k_z)\,,$$
$$\hat{P}\hat{T}\vec{s} = -\vec{s}\,. \tag{S6}$$

Therefore, the spin-polarized conduction channels do not exist in materials with $\hat{P}\hat{T}$ symmetry, such as centrosymmetric normal metal and some compensated antiferromagnets.

On the contrary, for crystals with violated $\hat{P}\hat{T}$ symmetry, the spin-polarized conduction channels are not forbidden. Specifically, $\hat{M}_z$ symmetry transforms the energy bands and the spin as follows:

$$\hat{M}_z E_n(\vec{k}_\parallel, k_z) = E_n(\vec{k}_\parallel, -k_z)\,,$$
$$\hat{M}_z s_x = -s_x\,,$$
$$\hat{M}_z s_y = -s_y\,,$$
$$\hat{M}_z s_z = s_z\,. \tag{S7}$$

This enforces $p_\parallel^x(\vec{k}_\parallel) = p_\parallel^y(\vec{k}_\parallel) = 0$ but allows $p_\parallel^z(\vec{k}_\parallel) \neq 0$, where $p_\parallel^i(\vec{k}_\parallel)$ is the spin polarization of the conduction channels at $k_\parallel$ along the quantization axis i ($i = x, y, z$). Similarly, a combination of the time reversal symmetry $\hat{T}$ and two-fold rotation about the z axis $\hat{C}_{2z}$ leads to

$$\hat{T}\hat{C}_{2z}E_n(\vec{k}_\parallel, k_z) = E_n(\vec{k}_\parallel, -k_z)\,,$$
$$\hat{T}\hat{C}_{2z}s_x = s_x\,,$$
$$\hat{T}\hat{C}_{2z}s_y = s_y\,,$$
$$\hat{T}\hat{C}_{2z}s_z = -s_z\,, \tag{S8}$$

which enforces $p_\parallel^z(\vec{k}_\parallel) = 0$ but allows finite $p_\parallel^x(\vec{k}_\parallel)$ and $p_\parallel^y(\vec{k}_\parallel)$.

We thus can conclude that if the transport direction is perpendicular to the $\hat{M}$ symmetry plane, the transport spin polarization is collinear to the current direction. On the other hand, if the transport direction is parallel to the $\hat{C}_2$ symmetry axis, the transport spin polarization is perpendicular to the current direction.

E. Electronic properties of RuO_2 with spin-orbit coupling

Figure S5 shows the calculated band structure and the Fermi surface of RuO_2 in the presence of spin-orbit coupling. We find that the spin-orbit coupling does not change much the band structure (compare Figs. S5a and Fig. 2b). Projecting the Fermi surface to the spin components (Fig. S5b-d) demonstrates that only a small portion of the Fermi surface has non-vanishing in-plane spin components. The most part of the Fermi surface is spin polarized along the z direction. Fig. S5e shows the calculated distribution of the conduction channels and their spin polarization in the $\vec{k}_{\parallel}$ plane indicating that the spin-polarized conduction channels are well preserved in the presence of spin-orbit coupling.

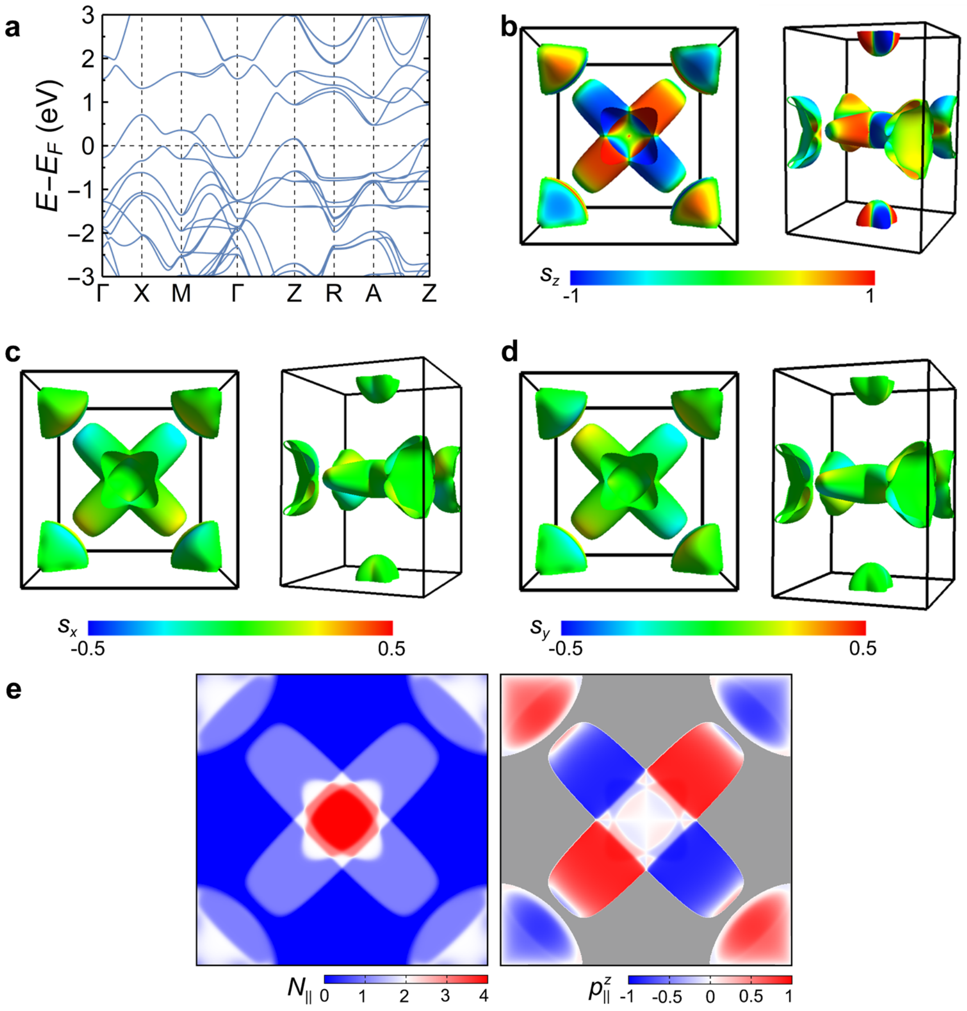

FIG. S5. Electronic and transport properties of RuO_2 with spin-orbit coupling. (a) The band structure of RuO_2. (b-d) The spin projected Fermi surface of RuO_2. (e) The number of conduction $N_{\parallel}$ (left) and their spin polarization $p_{\parallel}^{z}$ (right) in the 2D Brillouin zone. Grey color indicates regions where $N_{\parallel} = 0$ and hence $p_{\parallel}^{z}$ undefined.

F. Electronic properties of nonmagnetic TaN

Figure S6a shows the hexagonal structure of the nonmagnetic topological metal TaN [3-5]. It is seen that there is a strong band splitting near E_F due to spin-orbit coupling (Fig. S6b). One band crosses E_F near the K point, and four bands cross E_F along the Γ-A direction, resulting in large $N_{\parallel}(\vec{k}_{\parallel})$ at the center and corners of the 2D Brillouin zone (Figs. S6c,d). The mirror $\widehat{M}_z$ symmetry of the space group $P\bar{6}m2$ guarantees the spin polarization along the z direction. We find the conduction channels are almost fully polarized at the zone corners and are slightly spin polarized at the zone center (Figs. S6c,d). The total transport spin polarization is zero due to time reversal symmetry. Clearly, this nonmagnetic material supports an out-of-plane spin-neutral conductance through the conduction channels which are spin polarized along the current direction.

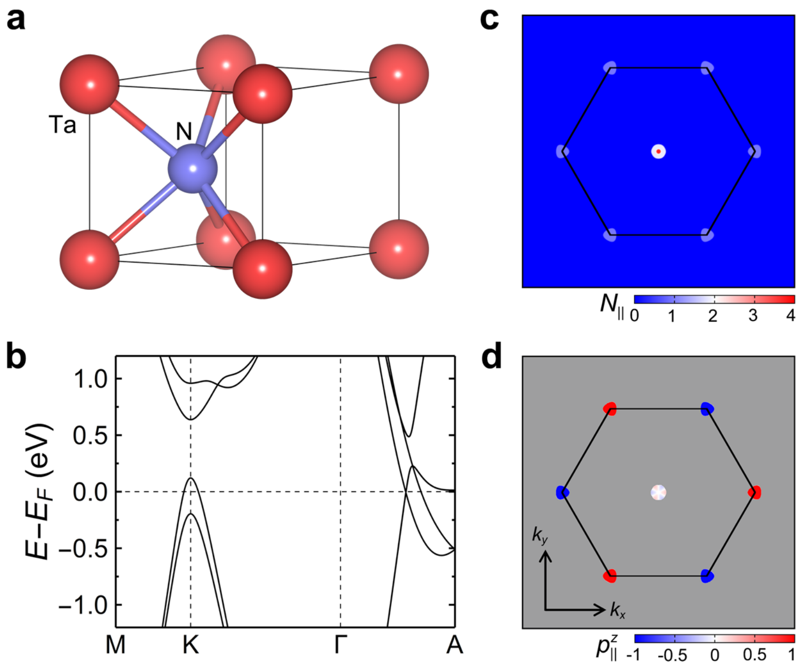

FIG. S6. (a,b) The atomic structure (a) and band structure (b) of TaN. (c,d) The number of conduction channels $N_{\parallel}$ (c) and their spin polarization $p_{\parallel}^z$ (d) in the 2D Brillouin zone (denoted by hexagons). Grey contrast indicates regions where $N_{\parallel} = 0$ and hence $p_{\parallel}^z$ undefined.

[1] A. Bose, N. J. Schreiber, R. Jain, D.-F. Shao, H. P. Nair, J. Sun, X. S. Zhang, D. A. Muller, E. Y. Tsymbal, D. G. Schlom, and D. C. Ralph, Tilted spin current generated by the collinear antiferromagnet RuO_2. *arXiv:*2108.09150 (2021).

[2] K. M. Schep, P. J. Kelly, and G. E. W. Bauer, Ballistic transport and electronic structure. *Phys. Rev. B* **57**, 8907 (1998).

[3] G. Brauer, E. Mohr, A. Neuhaus, and A. Skokan, θ-TaN, eine Hochdruckform von Tantalnitrid. *Monatsh. Chem.* **103**, 794 (1972)

[4] Z. Zhu, G. W. Winkler, Q. Wu, J. Li, and A. A. Soluyanov, Triple point topological metals. *Phys. Rev. X* **6**, 031003 (2016).

[5] H. Weng, C. Fang, Z. Fang, and X. Dai, Topological semimetals with triply degenerate nodal points in θ-phase tantalum nitride. *Phys. Rev. B* **93**, 241202 (2016).
